# Natural variation in social conditions affects male mate choosiness in the amphipod *Gammarus roeselii*

**DOI:** 10.1093/cz/zoab016

**Published:** 2021-02-24

**Authors:** Konrad Lipkowski, Sophie Steigerwald, Lisa M Schulte, Carolin Sommer-Trembo, Jonas Jourdan

**Affiliations:** Department of Wildlife/Zoo-Animal-Biology and Systematics, Institute for Ecology, Evolution and Diversity Goethe University Frankfurt, Max-von-Laue-Straße 13, Frankfurt am Main, D-60438, Germany; Department of Wildlife/Zoo-Animal-Biology and Systematics, Institute for Ecology, Evolution and Diversity Goethe University Frankfurt, Max-von-Laue-Straße 13, Frankfurt am Main, D-60438, Germany; Department of Environmental Science, Stockholm University, Svante Arrheniusväg 8, Stockholm, SE-11418, Sweden; Department of Wildlife/Zoo-Animal-Biology and Systematics, Institute for Ecology, Evolution and Diversity Goethe University Frankfurt, Max-von-Laue-Straße 13, Frankfurt am Main, D-60438, Germany; Zoological Institute, University of Basel, Vesalgasse 1, Basel, CH-4051, Switzerland; Department of Aquatic Ecotoxicology, Institute for Ecology, Evolution and Diversity, Goethe University Frankfurt am Main, Frankfurt am Main, Germany

**Keywords:** amplexus, Crustacea, local adaptation, mate choice, population density, sex ratio

## Abstract

The extent of male mate choosiness is driven by a trade-off between various environmental factors associated with the costs of mate acquisition, quality assessment and opportunity costs. Our knowledge about natural variation in male mate choosiness across different populations of the same species, however, remains limited. In this study, we compared male mate choosiness across 10 natural populations of the freshwater amphipod *Gammarus roeselii* (Gervais 1835), a species with overall high male mating investments, and evaluated the relative influence of population density and sex ratio (both affecting mate availability) on male mate choosiness. We investigated amplexus establishment after separating mating pairs and presenting focal males with a novel, size-matched female from the same population. Our analysis revealed considerable effects of sex ratio and (to a lesser extent) population density on time until amplexus establishment (choosiness). Male amphipods are able to perceive variable social conditions (e.g., sex ratio) and modify their mating strategy accordingly: We found choosiness to be reduced in increasingly male-biased populations, whereas selectivity increases when sex ratio becomes female biased. With this, our study expands our limited knowledge on natural variations in male mate choosiness and illustrates the importance of sex ratio (i.e., level of competition) for male mating decisions in natural environments. Accounting for variation in sex ratios, therefore, allows envisioning a distinctive variation of choosiness in natural populations and highlights the importance of considering social background information in future behavioral studies.

Traditionally, only females have been considered to be choosy during mate choice as they usually invest more resources into offspring than males (Darwin 1871; Bateman 1948; Trivers 1972). However, the important role of male mate choice is increasingly acknowledged (e.g., Bonduriansky 2001; Edward and Chapman 2011; Ah-King and Gowaty 2016; Schlupp 2018). Systems in which male mate choice occurs are often characterized by high male mating investment (e.g., due to costs associated with finding females and copulation) and high variance in quality among females (Edward and Chapman 2011). The extant of male mate choice is assumed to be driven by a trade-off between costs of being choosy (e.g., energy expenditure, Wong and Jennions 2003; opportunity costs, Barry and Kokko 2010) and net benefits from choosing a high-quality mate (Hubbell and Johnson 1987; Kvarnemo and Simmons 1999; Reading and Backwell 2007). Interestingly, this trade-off has shown to be context-dependent and is affected by various biotic and abiotic environmental factors; consequently, male choosiness varies among populations that are exposed to different conditions (Gwynne 1993; Wong and Jennions 2003; Dunn et al. 2008; Candolin and Salesto 2009; Lipkowski et al. 2019). For example, male poeciliid fish *Poecilia reticulata* are less choosy when exposed to high than low stream velocity (Head et al. 2010), and amphipod crustaceans show reduced choosiness in a high predation risk environment, *Gammarus duebeni* (Dunn et al. 2008).

However, social factors such as mate availability also affect male mate choosiness. For instance, theoretical models predict reduced choosiness under low mate availability (Bleu et al. 2012; Etienne et al. 2014; Courtiol et al. 2016). In terms of male mate choice, this can be expected in populations with low population density or male-biased sex ratio. In both cases, the probability to encounter a female mating partner is relatively low and the risk of remaining unmated upon rejection of a partner is high (Parker 1983). Hence, males from low-density populations and male-biased sex ratio are assumed to accept a broader range of female phenotypes. This hypothesis is supported by empirical evidence from insects (Shelly and Bailey 1992), crustaceans (Reading and Backwell 2007; Lipkowski et al. 2019), and fish (Berglund 1995; Svensson et al. 2010; Head et al. 2015). However, most studies investigated the influence of social parameters on male mate choice by artificially altering the respective social parameters after test animals have been introduced to the laboratory (see Table 2 in Ah-King and Gowaty 2016 for details). These studies mostly used a single population from which individuals were distributed to different social conditions. Thus, our knowledge about natural variation in male mate choosiness across different populations of the same species remains very limited. Furthermore, empirical studies in which the relative role of several social factors on male mate choosiness have been integrated and compared are scarce.

**Table 1.** Sampled populations and population parameters during the course of this study

Population	River	ASR (female/male)	ASR category	APD (individuals/h)	APD category
K1	Kinzig	1.8	F	300	High
S	Schwarzbach	0.3	M	134	Medium
U	Ulmbach	1	F/M	60	Medium
Sa	Salz	3.3	F≫	39	Low
Br1	Bracht	3.2	F≫	640	Very high
Br2	Bracht	1.6	F	86	Medium
K2	Kinzig	1.3	F	135	High
G1	Gründau	4.6	F≫	72	Medium
G2	Gründau	4	F≫	2400	Very High
K3	Kinzig	10.5	F≫	36	Low

APD categories (low *n* = 1–50, medium *n* = 51–100, high *n* = 101–500, very high *n* ≥ 501).

ASR categories (# adult females/# adult males: M = male-biased <1, F/M = sex equilibrium, F = female-biased 1,1–3, F**≫** = strong female-biased >3).

**Table 2.** Descriptive of the *event history analysis* of amplexus establishments according to APD and ASR

APD							ASR						
APD Category	N	Events	Ratio [%]	75% [min]	Median [min]	25% [min]	ASR Category	N	Events	Ratio [%]	75% [min]	Median [min]	25% [min]
Low	40	29	72.5	3.4	6.5	120	M	30	28	93.3	1.2	3.0	12.2
Medium	68	54	79.4	2.1	5.3	34.4	F/M	22	17	77.3	2.1	5.4	46.3
High	81	73	90,1	1.4	5.4	17.2	F	73	62	84.9	2.4	5.5	21.4
Very high	60	51	85	3.5	7.5	28	F≫	124	100	80.6	3.3	7.0	45.4
Overall	249	207	83.1	2.3	5.6	32.2							

APD categories (low *n* = 1–50, medium *n* = 51–100, high *n* = 101–500, very high *n* ≥ 501).

ASR categories (# adult females/# adult males: M = male-biased <1, F/M = sex equilibrium, F = female-biased 1,1–3, F**≫** = strong female-biased >3).

The observation took place for a maximum of 2 h. After that amplexus establishment was considered unsuccessful. Every event resembles an amplexus establishment.

In this study, we compared male mate choosiness across 10 natural populations of the freshwater amphipod *G. roeselii* (Gervais 1835) and evaluated the relative influence of 2 social factors on choosiness: population density and sex ratio. Male mating costs in amphipods are high due to prolonged precopulatory mate guarding (Hynes 1955; Birkhead and Clarkson 1980; Ward 1984; Elwood and Dick 1990). The mate guarding process begins with the formation of a so-called amplexus pair where the male grabs the female with its 1st gnathopods while being on the back of the female (Borowsky 1984; Conlan 1991). Males are mainly responsible for locomotion of amplexus pairs and effectively carry the females during precopula (Adams and Greenwood 1983). The amplexus lasts several days or weeks (Hynes 1955; Birkhead and Clarkson 1980; Sutcliffe 1992; Dick and Elwood 1996; Jormalainen 1998; Hume et al. 2002) and ends once the female molts and lays eggs for the male to fertilize (Sutcliffe 1992; Jormalainen 1998). This guarding behavior is usually considered as a male competitive strategy that may have evolved under high male–male competition for females (Parker 1974; Grafen and Ridley 1983). However, it represents a trade-off and comes with the costs of reduced feeding abilities (Robinson and Doyle 1985), increased risk of predation due to being a larger more visible target for predators with reduced escape performance (Strong 1973; Ward 1986; Cothran 2004) and an increased energy expenditure (Elwood and Dick 1990; Plaistow et al. 2003; but see Iltis et al. 2017). *Gammarus roeselii* (Gervais 1835) is an excellent model organism for our research question for the following reasons: First, due to immense costs of mate guarding, male mate choice plays a crucial role in this system (Birkhead and Clarkson 1980; Dunham et al. 1986; Elwood et al. 1987; Dick and Elwood 1989; Dick 1992; Jormalainen 1998; Kelly et al. 2001; Bollache et al. 2002). Second, amphipod populations show considerable natural interpopulation variation in population density (Cooper et al. 2012; Leite et al. 2014; Jourdan et al. 2019; Lipkowski et al. 2019) and sex ratio (from male-biased to strongly female-biased; Helan et al. 1973; Dick and Elwood 1996; Prato and Biandolino 2003; Jourdan et al. 2019); third, males were shown to be able to assess the level of intrasexual-competition (i.e., sex ratio/male density) and to evaluate differences in female quality (Ward 1983; Hunte et al. 1985; Elwood et al. 1987; Dick and Elwood 1989, 1996; Dunham and Hurshman 1990).

Here, we investigate the degree of male mate choosiness of 10 populations which naturally vary in population density and sex ratio. We used a 2-step analytical approach, in which we first applied an *Event History Analysis* to assess whether the 2 independent factors influence the time until and ratio of amplexus establishments. Afterward, we applied general linear models (GLMs) to determine which of the independent factors had the best explanatory value in predicting the median time and probability of amplexus establishment. We predict a higher degree of male mate choosiness in populations with 1) higher densities and 2) female-biased sex ratios (i.e., higher density of potential mates).

## Materials and Methods

### Study organism and sampling sites

*Gammarus roeselii* was described by Gervais in 1835 from a river near Paris (France; Karaman and Pinkster 1977), but actually originates from the Balkan region (Jażdżewski 1980; Grabowski et al. 2017; Csapó et al. 2020). Nowadays, it is known that *G. roeselii* is a species complex of which only one genetic lineage has colonized Central Europe (Csapó et al. 2020). Our study sites are situated in the Kinzig catchment, a tributary of the river Main (Supplementary Figure S1; Supplementary Table S1). A previous study confirmed that only one genetic lineage of *G. roeselii* is present in this area (Weigand et al. 2020), but at the same time, there are considerable differences in density and sex ratio between populations (Jourdan et al. 2019), rendering it an excellent model system for the investigation of social factors on male mating decision in amphipods. We collected animals using a “kick-and-sweep” technique (Barbour et al. 1999; Meier et al. 2006) by 2 people during a predefined time period of 60 min in an area of about 25 m^2^. On pebbly and rocky ground, we turned stones by hand and wiped animals from the stone surface into the net. Additionally, we carefully moved roots and aquatic plants that might serve as shelter for amphipods. We found pronounced differences in population densities ranging from 36–2,400 individuals per time effort collecting (2 persons/60 min) and predominant sex ratios ranging from male to heavily female-biased in sampled populations (Table 1). These results indicate pronounced differences in population density that appear to be stable over time (Jourdan et al. 2019).

### Maintenance conditions

We collected individuals for our behavioral tests in August 2019. All individuals were transferred into well-aerated cooling boxes filled with water from the collection site and brought within 1 h to the animal maintenance facilities of the Goethe University of Frankfurt. We maintained individuals separated by population in plastic aquaria (20 × 40 cm, water level 17 cm) containing stream water from the respective sampling site in climate chambers (KK2, THERMOTEC Weilburg GmbH & Co. KG, Weilburg, Germany). We gradually acclimated them to the maintenance temperature (10°C) and the test medium SAM-5S, prepared according to Borgmann (1996). This test medium is commonly used in amphipod behavioral studies to provide standardized maintenance and test environment (e.g., Feckler et al. 2012; Bundschuh et al. 2020). The acclimatization to the test medium was done by exchanging the water from the sampling sites with the SAM-5S medium via water exchanges over the course of 2 consecutive days (50% each day). Aquaria were equipped with air stones, securing continuously high oxygen contents, small stones, and leaves from the respective sampling sites to provide shelter and food. Individuals were additionally fed with a small amount of TetraMin Flakes (Tetra GmbH, Melle, Germany) ad libitum. The respective number of individuals per liter in the maintenance aquaria reflected the differences in actual population densities among populations. Males could freely choose from the pool of females inside the tanks and form amplexus pairs. We gave all test subjects 2 days for acclimatization before we randomly collected amplexus pairs for the behavioral tests.

### Environmental population parameters

To investigate the extent to which population density and sex ratio influence male mate choosiness, we assessed both parameters for each sampled population. To this end, all individuals that were not used for the behavioral tests (i.e., all remaining individuals from maintenance tanks) were preserved in 70% ethanol for body size and sex determination. Amphipods exceeding a length of 10 mm were considered to have reached sexual maturity (Macneil and Platvoet 2013), and were used to ascertain adult population density (APD = total number of adult individuals in maintenance tanks) and adult sex ratio (ASR = total number of adult females/total number of adult males). Individuals were sexed according to external sexual characteristics: males were identified by the presence of genital papillae and Oostegites or eggs in the brood pouch for females (Jourdan et al. 2019).

### Behavioral experiments—assessment of choosiness

We conducted behavioral tests in August 2019 to investigate the extent to which social population parameters influence male mate choosiness. To this end, we measured time until establishment of an amplexus pair after having separated the focal male from the female it had chosen initially (Elwood et al. 1987; Dick and Elwood 1989) and instead offering a novel, size-matched female (i.e., size-matched between the initial and the secondary female) from the same population, following Lipkowski et al. (2019). Our experimental design gave males ample opportunity to find their novel mate as individuals could easily swim through the small test area within several seconds to few minutes, consequentially resulting in frequent random tactile encounters between males and females. This minimized potential influences of female escape behavior (Sparkes et al. 2000; Bisazza et al. 2001), as well as potential variations in mate finding (Jones and Culver 1989), and locomotor abilities between populations (Bell and Stamps 2004; Dingemanse et al. 2007; Archard and Braithwaite 2011; Sommer-Trembo et al. 2017).

We placed *N *=* *249 amplexus pairs into individual glass beakers (diameter: 6.5 cm), filled with 200 mL of SAM-5S Medium, and gently separated the pair by briefly transferring it onto a wet piece of tissue, upon which the amplexus pair separated voluntarily. This approach is assumed to be the least invasive form of separating amplexus pairs (Dick and Elwood 1989). We visually size-matched body size between the initial and the secondary female. Upon completion of the behavioral trials, body sizes were determined to the closest 10th of a millimeter using a multizoom macroscope (Nikon AZ 100, Nikon GmbH, Düsseldorf, Germany) and an attached Nikon DS-Fi1 camera (Nikon GmbH, Düsseldorf, Germany). We used the software NIS-Elements BR 3.2 (Nikon GmbH, Düsseldorf, Germany) for all measurements of linear distances (millimeter). We determined the distance from the anterior margin of the head to the posterior margin of the telson as a measure of body size (Nahavandi et al. 2011). Mean (± SD) male body size was 13.8 ± 1.7 mm; mean female body size was 11.5 ± 1.6.mm. The average size difference between initially preferred and experimentally-offered novel females was 1.1 ± 1.0 mm.

The novel female stemmed from another experimentally separated amplexus pair, such that we could make sure that all females were in a similar reproductive state (i.e., before molt and sexually attractive to males). After the novel female was introduced on the side opposite to the focal male, we observed both individuals for 120 min. We measured the time until amplexus establishment and also noted down how many individuals did not form an amplexus within these 120 min. The applied period of observation time was chosen since a previous study showed that in this experimental setup most amplexus establishments occurred within the first 2 h (Lipkowski et al. 2019). Upon completion of the amplexus establishment tests, amplexus pairs were separated again and preserved in 70% ethanol with their initially chosen mate from the maintenance tank for subsequent measurements mentioned above.

### Statistical analyses

#### Amplexus establishment

All statistical analyses were conducted using SPSS 27 (SPSS Inc., Chicago, IL) and GraphPad Prism for visualization of results (Version 5.01, GraphPad Software). We used *Event History Analysis* (a.k.a. *Survival Analysis*, Kaplan–Meier Method) to analyze the time of amplexus establishment in *G. roeselii* populations. Therefore, we categorized populations according to APD and ASR into 4 categories each. APD was categorized as low (1–50), medium (51–100), high (101–500), or very high (>500). ASR was categorized as male-biased (<1; M), sex equilibrium (=1; F/M), female-biased (1.1-3; F), and strongly female-biased (>3; F≫). We used “time until amplexus establishment” as the dependent variable in a Kaplan–Meier Survival Analysis to test for population-wise differences in the time until amplexus establishment in relation to “APD” and “ASR” individually. Because our categories (factors; “APD,” “ASR”) follow a natural ordering (i.e., increasing sex ratio or densities for each factor level) we applied a log-rank test for trend to assess the statistical significance of the difference between the factor groups.

#### Social population parameters

We used GLMs in a population-specific analysis, to determine which of the explanatory variables “APD” and “ASR” best predicts male mating decisions. The dependent variables “time re-establishment” and “ratio amplexus establishment” derived from a preceding population-specific *Event History Analyses* (Supplementary Table S2; Supplementary Figure S2). The explanatory variables “APD” and “ASR” were log-transformed to filter off extreme values and improve linearity of the predictor variables. We included “APD” and “ASR” and their possible interaction terms as predictor variables in the initial model, but removed the interaction term if not significant at *P *≥* *0.05 (stepwise exclusion; see Supplementary Table S3 for nonsignificant effects). Assumptions of normality were assessed by testing for normality distribution (Shapiro–Wilk) and visual inspection of QQ-plots. Both, dependent variables as well as standardized model residuals of the applied final GLMs met the assumption of normality (Supplementary Table S4; see; Supplementary Figure S3 for Q–Q plots). Additionally, Pearson-correlation revealed no colinearity of our predictor variables (|r| = 0.07; *P *=* *0.985).

### Ethical approval

All applicable national and institutional guidelines for the care and use of animals were followed.

## Results

### Event history analysis—amplexus establishments

**Figure 1.**
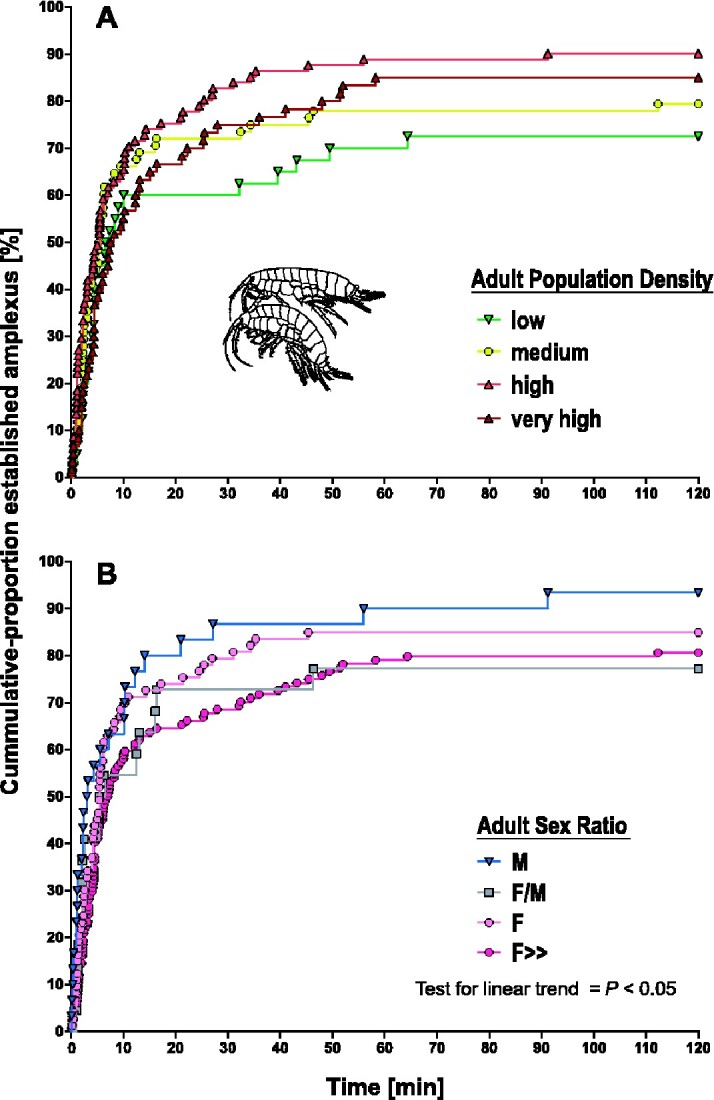
Visualization of event history analysis (*N *=* *249 amplexus pairs). Percentage of unpaired *G. roeselii* couples from 10 populations over the course of our experiment in relation to (**A**) APD and (**B**) ASR. Increments resemble amplexus establishments. Schematic view of an amplexus pair modified after Borowsky (1984).

We used *Event History Analysis* to test for differences between the time until amplexus establishment in dependency of “APS” and “ASR.” Descriptive results of amplexus establishments according to categorized social population parameters are summarized in Table 2. Log-rank test for trend (i.e., tests for a linear trend of factor levels) revealed a significant effect of “ASR” (log-rank test for trend, *χ*^2^ = 4.472, *P *=* *0.034) but not for “APD” (log-rank test for trend, *χ*^2^ = 1.222, *P *=* *0.269), indicating increasing ASR (female-biased populations) translate into longer time until amplexus establishments (Figure 1).

### GLM—socioenvironmental population parameters

**Figure 2.**
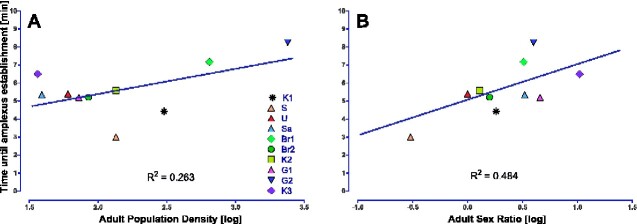
Visualization of significant main effects from the final GLM using “time until amplexus establishment” as a dependent variable (*N *=* *10 each) and log-transformed population parameters (**A**) APD and (**B**) ASR as predictor variables.

We used extracted median times and ratios of amplexus establishments for each individual population for our GLMs (Supplementary Table S2). Final GLMs (main effects) for “time until amplexus establishment” revealed significant effects of “APD” and “ASR” on extracted median time of amplexus establishment (APD: F_1,7_ = 7.573, MS = 5.050; *P *=* *0.028; ASR: F_1,7_ = 13.820, MS = 9.215; *P *=* *0.007). Subsequent visual examination reveals a positive association between “time until re-establishment” and the predictor variables “APD” (*R*^2^ = 0.263) as well as “ASR” (*R*^2^ = 0.484), indicating median time until reforming amplexus pairs increases with increasing population density and sex ratio; Figure 2). Final GLM for “ratio amplexus establishment” revealed no effect of “APD” or “ASR” on the ratio of amplexus establishment (APD: F_1,7_ = 2.590, MS = 162.414; *P *=* *0.152, ASR: F_1,7_ = 0.882, MS = 55.321; *P *=* *0.379).

## Discussion

In ththis study, we investigated the degree of male mate choosiness in 10 natural populations of *G. roeselii* which differed in 2 crucial social factors: population density and sex ratio. Our analyses revealed considerable effects of sex ratio and (to a lesser extent) population density on time until amplexus establishment (i.e., choosiness). Males from populations with a strongly female-biased sex ratio and from populations with higher population density took longer (i.e., were choosier) to establish an amplexus than males from less female-biased, sex equilibrium, male-biased populations, or populations with low population densities (Figure 2).

Choosiness is predicted to increase with increasing encounter rates of potential mates (Bleu et al. 2012; Etienne et al. 2014; Courtiol et al. 2016). Under low population density, the probability to encounter a suitable mate is decreased. Differences in population densities can be modulated by an array of biotic factors, including resource availability (Carbone and Gittleman 2002; Singh et al. 2016), and predation pressure (Heithaus 2004; Beauchamp et al. 2007; Heithaus et al. 2008), as well as abiotic environmental conditions (Leite et al. 2014). We found considerable differences in population density among our study populations. Congruent with our predictions, males from low-density populations were less choosy than males with ample opportunities to meet females in high-density populations. It should be pointed out though that despite extreme differences in population density (range: 36 individuals—2,400 individuals per unit effort sampling) the effect of population density on male mate choosiness was weaker than we expected. This could be explained by amphipods occurring in aggregations (Aumack et al. 2011; Vitaliano et al. 2013; Beermann et al. 2015) in some microhabitats (Korpinen and Westerbom 2010; Cooper et al. 2012) while being rather evenly distributed in others. Population density can therefore be low on average, but when animals encounter an aggregation of conspecifics, they can afford to be choosy. Therefore, even though population densities technically translate into encounter rates, the encounter probability of potential mates might still be high due to local aggregations.

Sex ratio had a strong effect on choosiness: males from increasingly male-biased populations established amplexus pairs significantly more readily than males from more female-biased populations. This is most likely due to a decreased pre-amplexus assessment of the offered female (Dick and Elwood 1989). Before mate guarding, male *Gammarus* spp. assess different parameters of female quality and try to increase their reproductive success, by choosing and investing in high-quality females to maximize their reproductive success (Elwood et al. 1987; Dick and Elwood 1989, 1990; Elwood and Dick 1990; Dick 1992; Kelly et al. 2001; Bollache et al. 2002). Our results are congruent with those of Dick and Elwood (1996) who sampled amplexus pairs from several *G. duebni celticus* populations differing in sex ratios and investigated the duration of mate guarding. They found that males from populations with balanced sex ratio form an amplexus up to 11 days longer than males from female-biased populations. The authors assumed that males under more intraspecific competition start to guard a female earlier. Our findings support this hypothesis and provide additional evidence that not only duration of mate guarding increase, but also the preamplexus assessment (i.e., choosiness) is reduced under higher male mate competition. The reproductive system in amphipods with brood-carrying females results in male-biased operational sex ratios (OSRs; Emlen and Oring 1977; Andersson 1994; Kvarnemo and Ahnesjo 1996; Székely et al. 2014) because the numbers of fertilizable females are much lower than the number of sexually-active males. This renders receptive females a scarce resource and results in general high male–male competition, which is thought to further intensify under increasingly male-biased sex ratios (Grafen and Ridley 1983; Elwood and Dick 1990; Weir et al. 2011). Reasons for a biased sex ratio in amphipods are manifold and still not fully understood (Dunn et al. 2020). Sometimes a greater susceptibility to mortality of 1 sex, due to differential sensitivity to adverse environmental conditions (e.g., Charlat et al. 2005), food availability (Trewick 1997; Kneib et al. 1997; Appleby et al. 1997), or predation mortality (Iwasa and Odendaal 1984; McKellar et al. 2009) can explain skewed sex ratios. Furthermore, some invertebrates are known for their environmental sex determination (determination in response to environmental conditions experienced by developing offspring; ESD; Adams et al. 1987; Korpelainen 1990), including sex determination cued by biotic (Bulnheim and Vávra 1968; Becheikh et al. 1998; Bouchon et al. 1998; Ironside et al. 2003; Moreau and Rigaud 2003) and abiotic factors (Bulnheim 1978; Ferguson and Joanen 1982; Naylor et al. 1988; Dunn et al. 2005; Warner and Shine 2008). Although not yet documented for *G. roeselii*, adaptive ESD might have evolved multiple times in amphipods (including closely-related ones; Bulnheim 1978; Korpelainen 1990; Dunn et al. 2005, 2020; Duffy et al., 2015). Our findings illustrate that male amphipods are able to perceive such changes in sex ratios and modify their mating strategy accordingly: reduced choosiness and extended guarding duration (Dick and Elwood 1996) is expected to occur in male-biased population, and increasing selectivity henceforth as the sex ratio becomes female biased. This is also reflected in the expression of crucial morphological characteristics: males under female-biased sex ratio have increased first antennae (relative to body size; Jourdan et al. 2019), indicating an increased investment into sensory traits used for mate assessment (Thornhill and Alcock 1983; Elgar et al. 2019; Lipkowski et al. 2019).

Since different sex ratios can have essential impact on behavioral (mating) decisions of amphipods, unraveling of factors that cause this immense variation in sex ratios across amphipod populations and species (Helan et al. 1973; Dick and Elwood 1996; Prato and Biandolino 2003; Jourdan et al. 2019) is an interesting field of future research. This is also linked to the question of how consistent sex ratios are over time. The consistency (i.e., predictability) of social conditions may impact to what degree heritability (e.g., Seghers 1974; Ariyomo et al. 2013; Dochtermann et al. 2014) and phenotypic plasticity (e.g., Daza-Bustamante et al. 2002; Hays et al. 2002; Sommer-Trembo et al. 2017) underlie the observed behavioral responses between populations. In the populations used for this study, our measurements of sex ratio, were similar to previous investigations (2017–2019; see Jourdan et al 2019), suggesting rather stable sex ratios over time, which renders rapid local adaptation based on heritable differences in choosiness among populations a possible explanation for our observations. However, sex ratios of many amphipod species are known to be variable (e.g., Helan et al. 1973; Naylor et al. 1988; Dick and Elwood 1996; Prato and Biandolino 2003) and our limited number of measurements on the same populations cannot rule out variation within or between years. A straightforward approach to investigate the relative contributions of genetic adaptation and phenotypic plasticity of male mate choosiness would be to conduct transgenerational experiments in the laboratory. The *Hyalella azteca* species complex (e.g., Weston et al. 2013) could be considered as a surrogate species, since these species are easier to maintain and breed. Altogether, our results identify mate-guarding amphipods as a promising model to further elucidate the underlying evolutionary phenomena shaping mate choice behavior under variable social conditions.

## Supplementary Material

zoab016_Supplementary_Data
