# Supplementary material for: Natural variation in social conditions affects male mate choosiness in the amphipod Gammarus roeselii
Source: Curr Zool. 2021 Feb 24;68(4):459–68. doi: 10.1093/cz/zoab016 (PMC9450172; doi:10.1093/cz/zoab016)
Supplement: zoab016_Supplementary_Data [file zoab016_supplementary_data.zip › Supplement.docx]

**Supplementary material**

**Natural variation in social conditions affects male mate choosiness in the amphipod *Gammarus roeselii***

Konrad Lipkowski^a,*^, Sophie Steigerwald^a,b^, Lisa M. Schulte^a^, Carolin Sommer-Trembo^c^, Jonas Jourdan^d^

^a^Department of Wildlife/Zoo-Animal-Biology and Systematics, Institute for Ecology, Evolution and Diversity Goethe University Frankfurt, Max-von-Laue-Straße 13, D-60438 Frankfurt am Main, Germany; ^b^ Department of Environmental Science, Stockholm University, Svante Arrheniusväg 8, SE-11418 Stockholm, Sweden; ^c^ Zoological Institute, University of Basel, Vesalgasse 1, CH-4051 Basel, Switzerland; ^d^Department of Aquatic Ecotoxicology, Institute for Ecology, Evolution and Diversity, Goethe University Frankfurt am Main,Frankfurt am Main, Germany

*Address correspondence to Konrad Lipkowski. Emails: [lipkowski@bio.uni-frankfurt.de](mailto:lipkowski@bio.uni-frankfurt.de)

Handling editor: Zhi-Yun JIA

Received on 30 January 2021; accepted on 18 February 2021

**Table S1.** Location of sampling sites in our study area of the Kinzig-catchment in the Main-Kinzig-Area in Hesse, Germany

| Population | River | Latitude | Longitude |
| --- | --- | --- | --- |
| K1 | Kinzig | 50°20'46.8"N | 9°33'07.4"E |
| S | Schwarzbach | 50°21'31.8"N | 9°33'07.0"E |
| U | Ulmbach | 50°23'26.1"N | 9°24'02.9"E |
| Sa | Salz | 50°24'59.9"N | 9°21'48.4"E |
| Br1 | Bracht | 50°22'37.2"N | 9°16'13.3"E |
| Br2 | Bracht | 50°16'40.9"N | 9°18'34.5"E |
| K2 | Kinzig | 50°12'31.2"N | 9°13'42.5"E |
| G1 | Gründau | 50°14'55.8"N | 9°09'20.2"E |
| G2 | Gründau | 50°12'46.7"N | 9°06'06.4"E |
| K3 | Kinzig | 50°09'09.4"N | 9°00'41.4"E |

**Table S2.** Descriptives of the *Event History Analysis* of amplexus establishments with individuals from all sampled populations. The observation took place for a maximum of two hours after that amplexus establishment was considered unsuccessful.

| **Population** |  | **Case Processing Summary** | | |  | **Amplexus establishment times** | | |
| --- | --- | --- | --- | --- | --- | --- | --- | --- |
|  |  | N | Censored | Separate [%] |  | Median [min] | IQR-75% [min] | IQR-25% [min] |
| K1 |  | 26 | 3 | 11.5 |  | 4.43 | 4.1 | 43.17 |
| S |  | 30 | 2 | 6.7 |  | 3 | 2.35 | 25.45 |
| U |  | 22 | 5 | 22.7 |  | 5.4 | 1.45 | 21.39 |
| Sa |  | 22 | 8 | 36.4 |  | 5.35 | 4.14 | 22.2 |
| Br1 |  | 30 | 6 | 20 |  | 7.17 | 2.2 | 34.35 |
| Br2 |  | 22 | 5 | 22.7 |  | 5.21 | 2.44 | 9.4 |
| K2 |  | 25 | 3 | 12 |  | 5.29 | 3.38 | 51.5 |
| G1 |  | 24 | 4 | 16.7 |  | 5.2 | 3.4 | 120 |
| G2 |  | 30 | 4 | 10 |  | 8.25 | 2.1 | 46.34 |
| K3 |  | 18 | 3 | 16.7 |  | 6.5 | 1.19 | 12.2 |

**Table S3.** Non-significant interaction terms that were removed from the final general linear models analysing the effect of our predictor variables APD (adult population density) and ASR (adult sex ratio) on median time and ratio of amplexus establishment. Terms were excluded step-wise based on *P*< 0.05.

|  | | *Time until amplexus establishment* | | | |  | *Ratio amplexus establishment* | | |
| --- | --- | --- | --- | --- | --- | --- | --- | --- | --- |
| Term | *df* | | *Mean Square* | *F* | *P* |  | *Mean square* | *F* | *P* |
| APD x ASR | 6 | | 1.732 | 3.541 | 0.109 |  | 189.460 | 4.557 | 0.077 |

**Table S4.** Test for normality (Shapiro-Wilk) of dependent variables and standardized model residuals of applied final general linear models using ‘time until amplexus establishment’ and ‘ratio amplexus establishment’ as dependent variables.

|  | *Dependent variable* | |  | *Model residuals* | |
| --- | --- | --- | --- | --- | --- |
|  | *df* | *P* |  | *df* | *P* |
| *Time until amplexus establishment* | 10 | 0.722 |  | 10 | 0.705 |
| *Ratio amplexus establishment* | 10 | 0.355 |  | 10 | 0.613 |

**
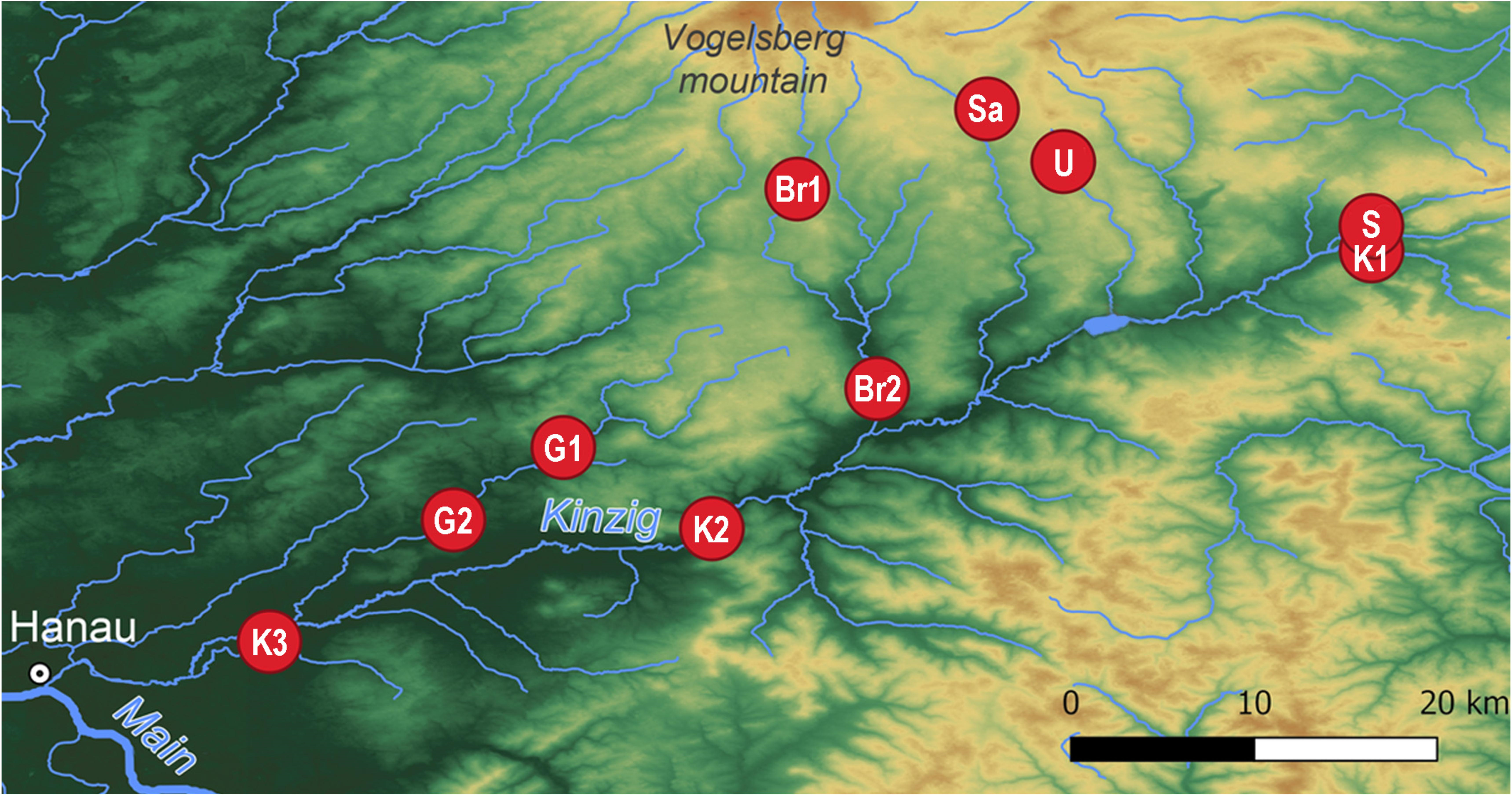
Figure S1.** Map of the study area and location of sampling sites in the Kinzig-catchment in the Main Kinzig-Area, Hesse, Germany.


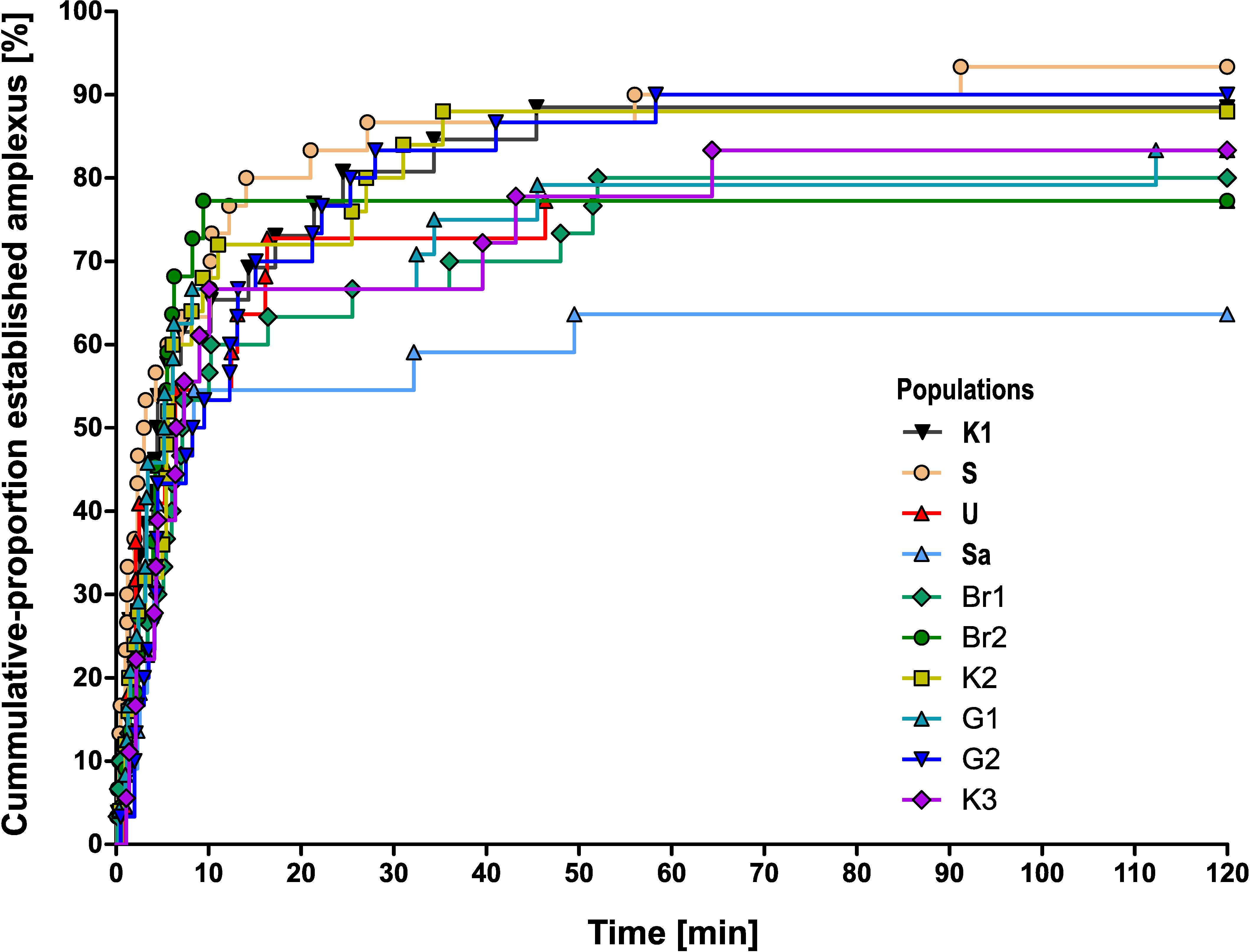


**Figure S2.** Visualisation of Event History Analysis Percentage of unpaired *Gammarus roeselii* couples from ten populations over the course of our experiment. Increments of the curve resemble amplexus re-establishments event.

**
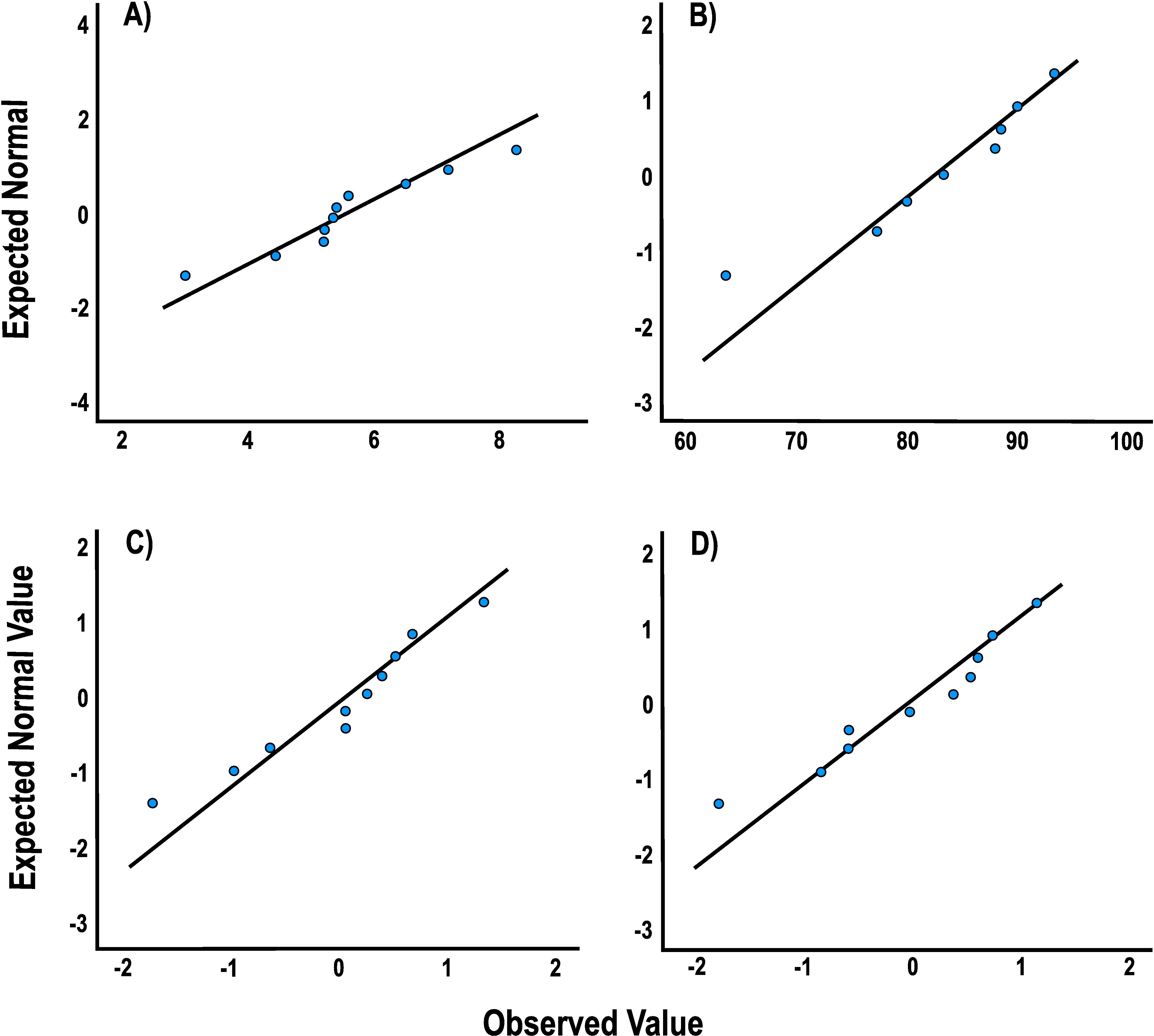
Figure S3.** Quantile – quantile (QQ) plots of dependent variables A) time until amplexus establishment, B) ratio amplexus establishment and model residuals of our general linear model with C) time until amplexus establishment and D) ratio amplexus establishment as dependent variables.
