# Supplementary material for: Natural variation in social conditions affects male mate choosiness in the amphipod Gammarus roeselii
Source: Curr Zool. 2021 Feb 24;68(4):459–68. doi: 10.1093/cz/zoab016 (PMC9450172; doi:10.1093/cz/zoab016)
Supplement: zoab016_Supplementary_Data [file zoab016_supplementary_data.zip › Supplement.pdf]

Supplementary material

Natural variation in social conditions affects male mate choosiness in the amphipod *Gammarus roeselii*

Konrad Lipkowski[a,*], Sophie Steigerwald[a,b], Lisa M. Schulte[a], Carolin Sommer-Trembo[c], Jonas Jourdan[d]

[a]Department of Wildlife/Zoo-Animal-Biology and Systematics, Institute for Ecology, Evolution and Diversity Goethe University Frankfurt, Max-von-Laue-Straße 13, D-60438 Frankfurt am Main, Germany; [b] Department of Environmental Science, Stockholm University, Svante Arrheniusväg 8, SE-11418 Stockholm, Sweden; [c] Zoological Institute, University of Basel, Vesalgasse 1, CH-4051 Basel, Switzerland; [d]Department of Aquatic Ecotoxicology, Institute for Ecology, Evolution and Diversity, Goethe University Frankfurt am Main,Frankfurt am Main, Germany

*Address correspondence to Konrad Lipkowski. Emails: lipkowski@bio.uni-frankfurt.de

Handling editor: Zhi-Yun JIA

Received on 30 January 2021; accepted on 18 February 2021

Table S1. Location of sampling sites in our study area of the Kinzig-catchment in the Main-Kinzig-Area in Hesse, Germany

Population	River	Latitude	Longitude
K1	Kinzig	50°20'46.8"N	9°33'07.4"E
S	Schwarzbach	50°21'31.8"N	9°33'07.0"E
U	Ulmbach	50°23'26.1"N	9°24'02.9"E
Sa	Salz	50°24'59.9"N	9°21'48.4"E
Br1	Bracht	50°22'37.2"N	9°16'13.3"E
Br2	Bracht	50°16'40.9"N	9°18'34.5"E
K2	Kinzig	50°12'31.2"N	9°13'42.5"E
G1	Gründau	50°14'55.8"N	9°09'20.2"E
G2	Gründau	50°12'46.7"N	9°06'06.4"E
K3	Kinzig	50°09'09.4"N	9°00'41.4"E

Table S2. Descriptives of the *Event History Analysis* of amplexus establishments with individuals from all sampled populations. The observation took place for a maximum of two hours after that amplexus establishment was considered unsuccessful.

Population	Case Processing Summary			Amplexus establishment times		
	N	Censored	Separate [%]	Median [min]	IQR-75% [min]	IQR-25% [min]
K1	26	3	11.5	4.43	4.1	43.17
S	30	2	6.7	3	2.35	25.45
U	22	5	22.7	5.4	1.45	21.39
Sa	22	8	36.4	5.35	4.14	22.2
Br1	30	6	20	7.17	2.2	34.35
Br2	22	5	22.7	5.21	2.44	9.4
K2	25	3	12	5.29	3.38	51.5
G1	24	4	16.7	5.2	3.4	120
G2	30	4	10	8.25	2.1	46.34
K3	18	3	16.7	6.5	1.19	12.2

Table S3. Non-significant interaction terms that were removed from the final general linear models analysing the effect of our predictor variables APD (adult population density) and ASR (adult sex ratio) on median time and ratio of amplexus establishment. Terms were excluded step-wise based on $P < 0.05$.

		Time until amplexus establishment			*Ratio amplexus establishment*		
Term	*df*	*Mean Square*	*F*	*P*	*Mean square*	*F*	*P*
APD x ASR	6	1.732	3.541	0.109	189.460	4.557	0.077

Table S4. Test for normality (Shapiro-Wilk) of dependent variables and standardized model residuals of applied final general linear models using 'time until amplexus establishment' and 'ratio amplexus establishment' as dependent variables.

	Dependent variable		*Model residuals*	
	df	*P*	*df*	*P*
Time until amplexus establishment	10	0.722	10	0.705
Ratio amplexus establishment	10	0.355	10	0.613

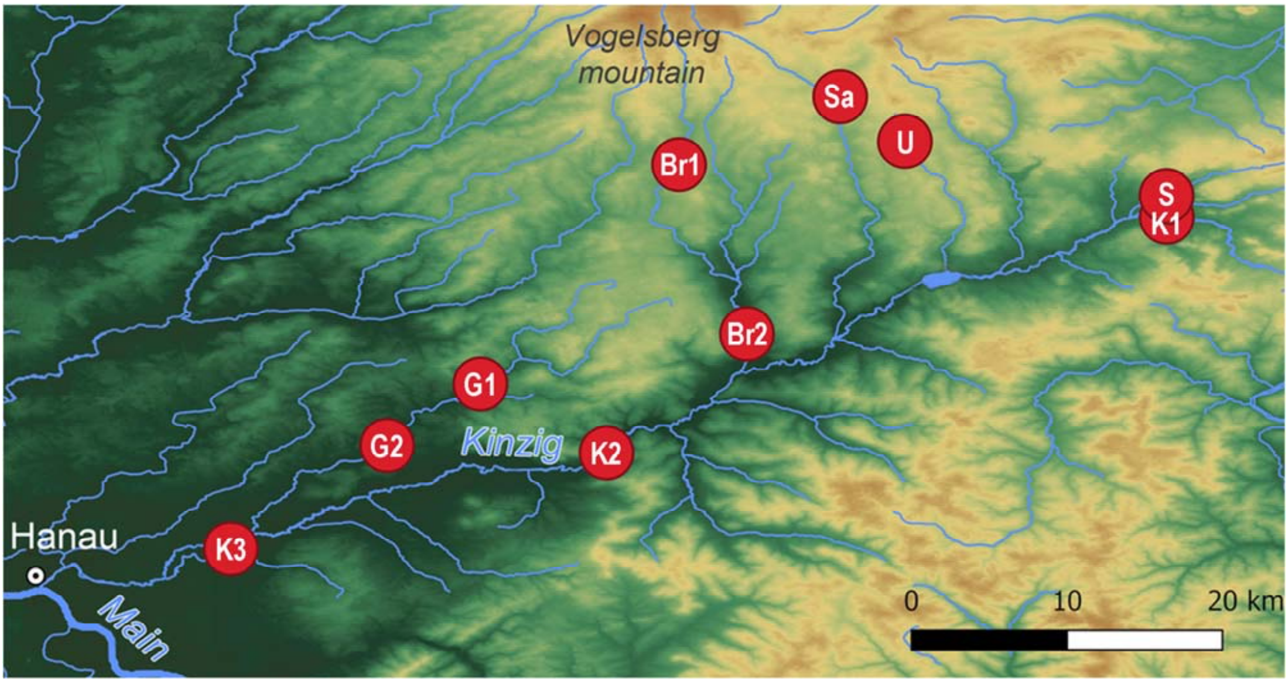

Figure S1. Map of the study area and location of sampling sites in the Kinzig-catchment in the Main Kinzig-Area, Hesse, Germany.

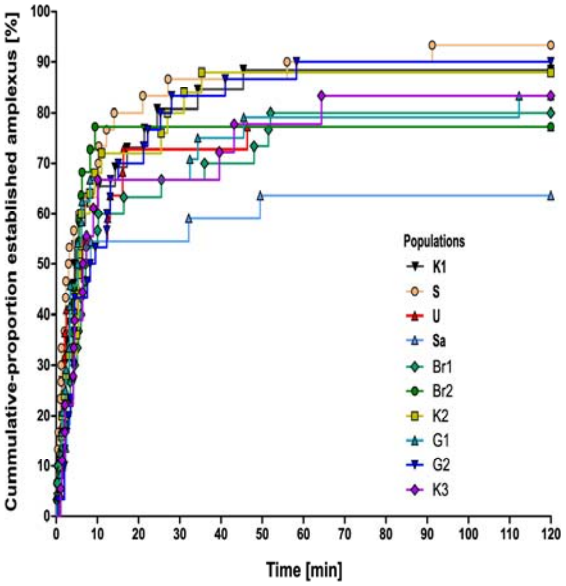

Figure S2. Visualisation of Event History Analysis Percentage of unpaired *Gammarus roeselii* couples from ten populations over the course of our experiment. Increments of the curve resemble amplexus re-establishments event.

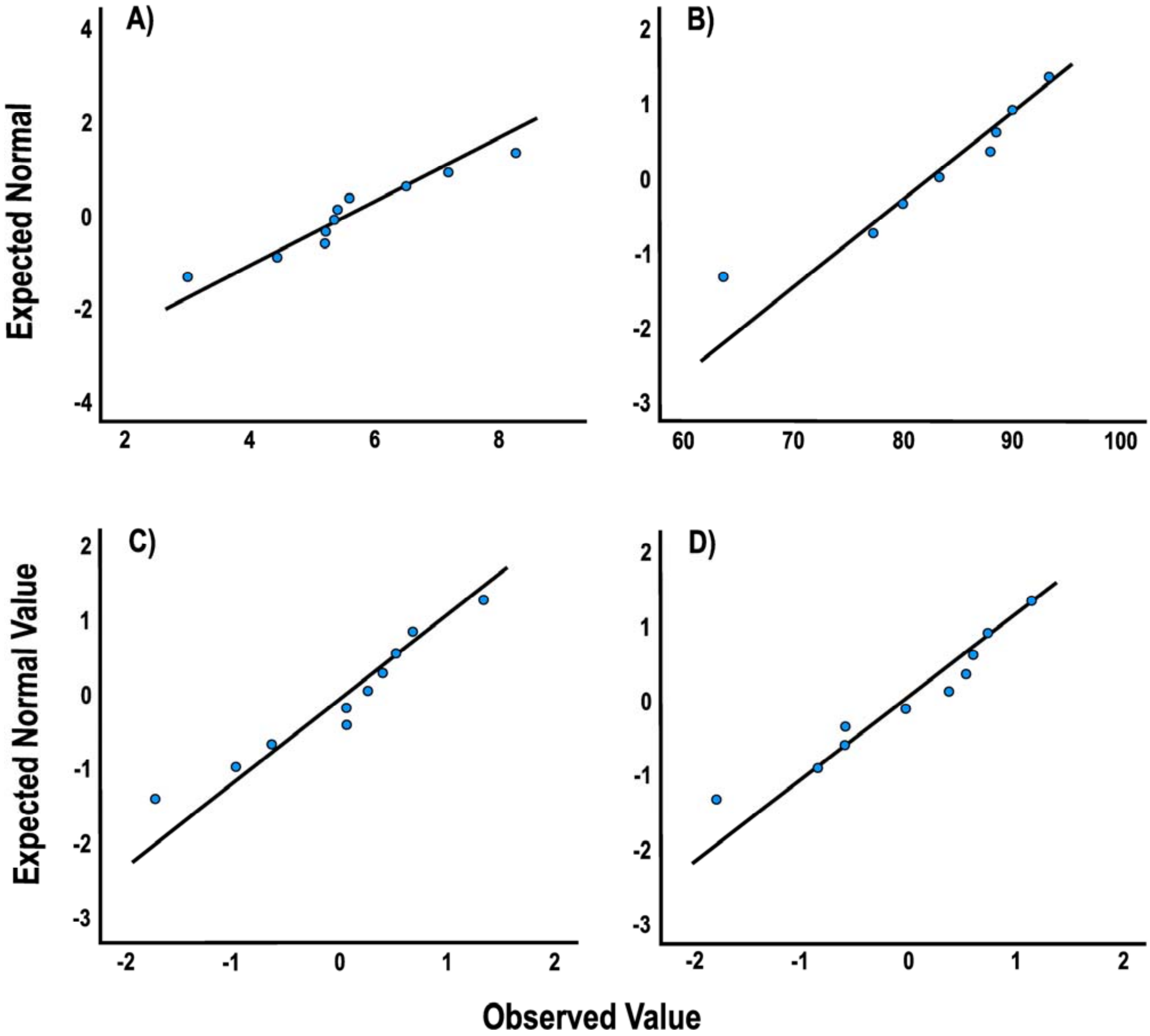

Figure S3. Quantile – quantile (QQ) plots of dependent variables A) time until amplexus establishment, B) ratio amplexus establishment and model residuals of our general linear model with C) time until amplexus establishment and D) ratio amplexus establishment as dependent variables.
